# Regulatory CAR-T cells in autoimmune diseases: Progress and current challenges

**DOI:** 10.3389/fimmu.2022.934343

**Published:** 2022-08-10

**Authors:** Tobias Riet, Markus Chmielewski

**Affiliations:** Department I for Internal Medicine, Center of Molecular Medicine Cologne (CMMC), University Hospital of Colgne, Cologne, Germany

**Keywords:** regulatory (Treg) cell, chimeric antigen receptor (CAR), autoimmune diseases, adoptive therapy, immunosuppressive therapy

## Abstract

CAR (Chimeric Antigen Receptor) T-cell therapy has revolutionized the field of oncology in recent years. This innovative shift in cancer treatment also provides the opportunity to improve therapies for many patients suffering from various autoimmune diseases. Recent studies have confirmed the therapeutic suppressive potential of regulatory T cells (Tregs) to modulate immune response in autoimmune diseases. However, the polyclonal character of regulatory T cells and their unknown TCR specificity impaired their therapeutic potency in clinical implementation. Genetical engineering of these immune modulating cells to express antigen-specific receptors and using them therapeutically is a logical step on the way to overcome present limitations of the Treg strategy for the treatment of autoimmune diseases. Encouraging preclinical studies successfully demonstrated immune modulating properties of CAR Tregs in various mouse models. Still, there are many concerns about targeted Treg therapies relating to CAR target selectivity, suppressive functions, phenotype stability and safety aspects. Here, we summarize recent developments in CAR design, Treg biology and future strategies and perspectives in CAR Treg immunotherapy aiming at clinical translation.

## Introduction

Regulatory T cells (Treg) are a critical CD4 T cell subset involved in the control of immune-tolerance. Tregs regulate immune-homeostasis and limit immune activation mediated by proinflammatory activities of CD4+ and CD8+ T cells, natural killer (NK) cells, and antigen-presenting cells (APC). Furthermore, Treg cells harbor powerful suppressive potential to promote tissue repair (1, 2) and modulate metabolic regulation (3). Thus, defects in Tregs that induce an imbalance of immune regulation often lead to autoimmune disorders (4). On the other hand, the involvement of immunosuppressive regulatory T cells plays a central role in tumor progression in both human and mice (5, 6). Accordingly, numerous groups have demonstrated an increased persistence of regulatory T cells in tumor tissue and surrounding tumor microenvironment (TME) (7). A continuous high expression of the transcription factor forkhead box protein P3 (FOXP3) in Treg cells is considered to be essential for their suppressive activity (8). Two main groups of FOXP3 Treg cells exist: natural Treg (nTreg) cells that develop in the thymus and represent a professional, stable T-cell lineage and peripherally induced Treg cells (pTreg) that differentiate from naïve CD4+ T cells in the periphery after antigen receptor stimulation in the presence of transforming growth factor beta (TGF-beta) (9). Lyon and colleagues demonstrated the importance of FOXP3 for Treg cell functions by their finding that mutation in the *FoxP3* locus in mice leads to Treg dysfunction and severe autoimmunity (10). Bennett, as well as Wildin and colleagues, confirmed that IPEX syndrome is the human equivalent of the scurfy mouse phenotype by identifying mutations in the *FOXP3* gene, the human homolog of the mouse gene *FoxP3* (11, 12). Furthermore, Barnes and colleagues demonstrated that CTLA-4 crosslinking on the surface of regulatory T cells in the presence of TCR signal enhanced the generation of FOXP3+ T cells (13). Moreover, CTLA-4 engagement changed the effect of CD28 crosslinking from inhibiting to promoting FOXP3 expression (13). As demonstrated by Read and colleagues (14), inhibition of CTLA-4 receptor in mouse abrogates Treg cell-mediated protection and, similarly, blocking of CTLA-4 in cancer patients improves anti-tumor immune response but also boosts autoimmunity (15). Furthermore, human Treg cells affect surrounding immune cells by releasing immunosuppressive cytokines including TGF-beta, IL-10, and IL-35 (16). Because of their proven immunomodulatory properties, Treg cells became an attractive therapeutic tool for treating autoimmune diseases and modulating or preventing transplant rejection and graft vs. host disease (GvHD). In recent years, several phase I clinical trials aiming to investigate safety and feasibility of a Treg-based therapy were conducted, thereby revealing chances and challenges of this immunotherapeutic strategy (see **Table 1**). For example, autologous *ex-vivo* expanded polyclonal Tregs have been transferred in 2009 to respective recipients who suffered from either acute or chronic GvHD (21). Since then, the therapeutic potential of Treg cells was gradually widened to autoimmunological diseases such as Type 1 diabetes (22), cutaneous lupus (23), autoimmune hepatitis (24) or Crohn’s disease (25), and to prevent rejection in solid organ transplantation (26), but the treatment modalities changed only slightly. Novel technologies to alter the genome of the Treg cells might enhance functional activity, stability, persistence and antigen specificity, and could broaden the therapeutic capacity of this promising immunotherapeutic strategy. Numerous preclinical studies have revealed that antigen-specific or redirected Treg cells are superior as compared to classical polyclonal Treg cells in diverse mouse models (27–30). Redirected Treg cells predominantly localize at the site of target antigen expression, thereby reducing the risk of systemic immunosuppression. Thus, CAR or TCR redirected Treg cells seem to be more effective and safer than polyclonal Tregs cells. In addition, advances in the field of Treg biology open up new possibilities to generate Tregs from naïve T CD4+ T cells through targeted modifications, such as induction of FOXP3 expression (30, 31).

**Table 1:** Clinical trials that use or affect regulatory T cells.

A) ongoing/recruiting/planned clinical trials					
	all diseases	GvHD	transplantation	autoimmune diseases	other*
		
all interventions	109	14	23	55	17
polyspecific Tregs	29	7	10	10	2
redirected Tregs	3	–	2** (17, 18)	–	1
converted Tregs	1	–	–	1	–
*in vivo* Treg stimulation	76	7	11	44	14
B) completed clinical trials					
** **	all diseases	GvHD	transplantation	autoimmune diseases	other*
		
all interventions	160	12	19	102	27
polyspecific Tregs	16	5	7 (19)	2 (20)	2
redirected Tregs	–	–	–	–	–
converted Tregs	–	–	–	–	–
*in vivo* Treg stimulation	144	7	12	100	25

*other: allergy, infections, cancer, pregnancy, other rare diseases etc.

** HLA-A2 specific CAR Tregs

(source: clinicaltrials.gov, search term “regulatory t cells”, record date 2022-06-28)

## Treg cells: Phenotype and function

Thymically derived FOXP3+ regulatory T cells (nTreg, formerly tTregs) constitute a unique T cell lineage that is essential for maintaining immune tolerance to self as well as innocuous environmental antigens and intra-tissue immune homeostasis. These cells develop from antigen-unexperienced naïve Tregs with expression of CD4+, CD25+, CD127dim/–, CD45RA+ (32) to natural Tregs (nTreg) which account for 1-4% of all white blood cells and express CD4+, CD25hi, CD127dim/– Helios+. However, FOXP3 can also be turned on in conventional T cells with effector functions (Teff) as consequence of antigen exposure in the periphery, under both non-inflammatory and inflammatory conditions. These so-called peripheral Treg cells (pTreg, formerly induced Tregs/iTregs) that involve both CD4+ and CD8+ pTreg cells are characterized by CD4+/CD8+, CD25hi, CD127dim/– expression and participate in the control of immunity at sites of inflammation. Although phenotypically and functionally similar, nTregs can be clearly discriminated from pTregs by their stable epigenetic modification of the Treg-specific demethylated region (TSDR) of the *FOXP3* gene (33). Peripherally induced Tregs are also considered to be negative for Helios expression, in contrast to nTreg, but this is still controversially debated in the field (34–41). The both, nTreg and pTreg can control inflammation/immunity by multiple mechanisms, such as i) competition with Teff for IL-2, ii) through cAMP-mediated immunosuppression, iii) adenosine production *via* the ectoenzymes CD39 and CD79, iv) secretion of inhibitory cytokines (e.g. IL-10, TGF-beta, IL-33, IL-34), and v) cytolysis of Teff *via* granzyme/perforin-dependent mechanisms (42, 43).

Despite some other common specific cell surface markers like GITR and CTLA-4 (44), many additional molecules describe various subpopulations of Treg cells like CD39 (45, 46) and CD49d (47, 48). Although a stable and high FOXP3 expression seems to be crucial for Treg cell function, recent data point to a limited effect on functional characteristics of FOXP3-ablated Tregs (49). There is increasing evidence that intra-tissue antigen-driven activation and inflammation promotes FOXP3 instability even in Treg that expressed high amounts of FOXP3 before. Key factors of instability seem to be lack of IL-2, inflammatory cytokines (Stat3, NFkappaB pathways) and activation of certain costimulatory molecules, so called switch-points (50, 51). Diminished number and/or function of Treg, e.g. by malformation in IPEX syndrome, plus a misbalanced Teff/Treg ratio at the site of inflammation result in unwanted inflammation/immunity associated with autoimmunity, autoinflammation, and disturbed regeneration from trauma and ischemia/reperfusion (52). Consequently, agonistic targeting of Treg is a promising therapeutic option to combat undesired inflammation/immunity in a broad range of medical indications. Although *in vivo* Treg induction/expansion approaches such as blocking costimulatory signals during antigen exposure, tolerogenic dendritic cells, tolerogenic peptide vaccination, and low-dose IL-2 show some efficacy in preclinical models and first in human trials, their efficacy is limited and some adverse effects can be observed (53, 54). In many preclinical models, the adoptive transfer of Treg seems to be more effective but recent technological advances to isolate and expand human Treg under GMP compliant conditions now allow adoptive Treg therapy to be more and more introduced to the clinic, thereby opening up new opportunities.

Based on the analysis of very recent preclinical and clinical studies on 1st-generation Treg products, the main objectives to develop next-generation Treg approaches with enhanced efficacy are:

- To improve the antigen specificity of Treg products by generating i) Chimeric Antigen Receptor-expressing Treg (CAR-Treg) (27, 55–59) or ii) specific T cell receptor-expressing Treg (TCR-Treg) (60–65).

- To stabilize the *in vivo* suppressive function of Treg by i) epigenetic FOXP3 gene modification (66–69), ii) CRISPR/Cas9 mediated knocking-out of switch-point-receptors whose activation leads to loss of Treg function or even to the switch to effector cells (70, 71), or iii) mitochondrial modification (72–74) iv) exploring CD8+ Treg as an alternative and/or complementary to CD4+ Treg (75–81), v) inducing resistance to immunosuppressive drugs using CRISPR/Cas9 mediated knocking-out of their target molecules (82, 83), vi) insertion of an additional *FOXP3* gene cassette, which also works as a safety mechanism to prevent Treg to Teff conversion (56, 84, 85), vii) development of Treg supporting IL-2 muteins or orthogonal IL-2 pairings (86–89).

## CAR-History

**Figure 1:**
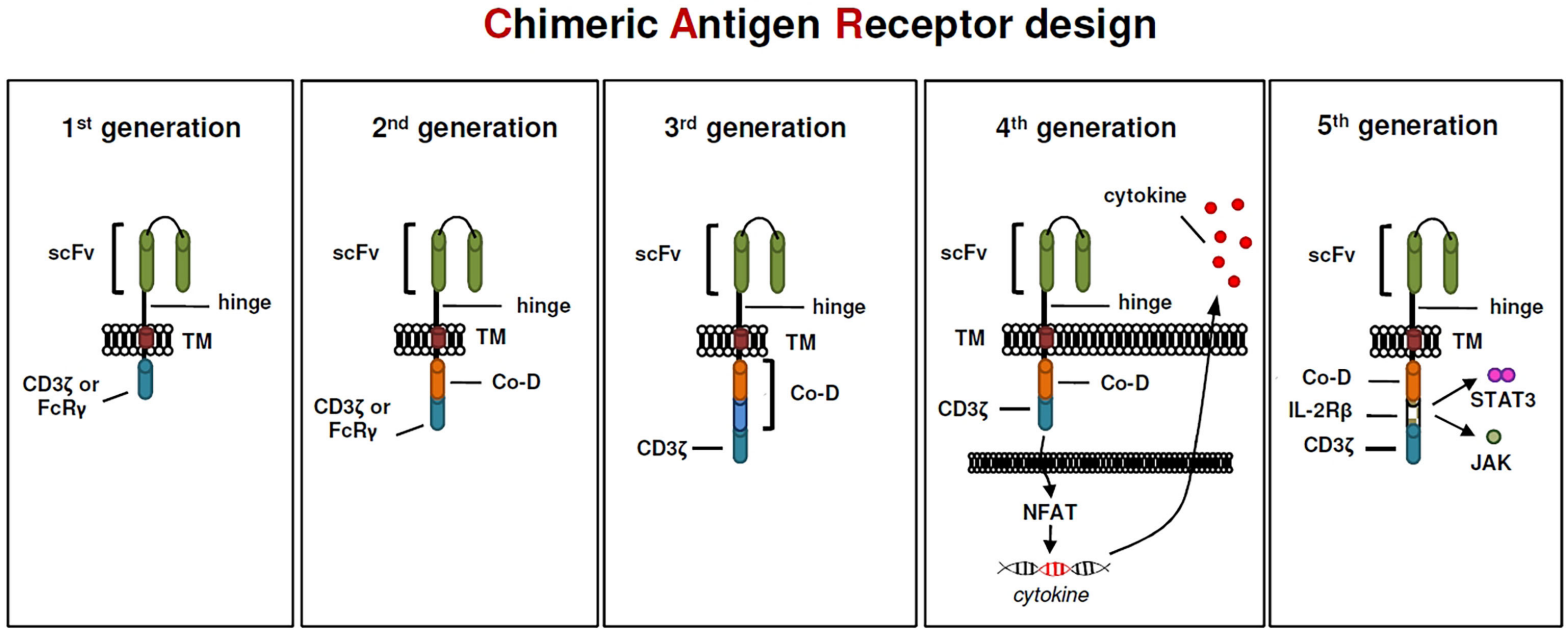
The evolution of CARs.

Although, to many of us, it seems that CAR T cells have only recently entered the world stage, their origin dates back forty years. In 1982, almost unnoticed by the research community, Zelig Eshhar demonstrated for the first time a CAR prototype, so called “T-body”, that conceptually has a lot in common with today’s CAR constructs (90). The first CAR constructs designed by Eshhar and colleagues contained a TNP-specific scFv binding domain linked to either the CD3zeta or the FcRgamma signaling domain for T cell activation (90). These first-generation CARs (**Figure 1**) did not provide an additional costimulatory domain that is essential for full T-cell activation. CAR constructs designed this way were still functional because T cells could be able to replace the missing coactivating signal (also known as signal 2) *via* the endogenous CD28-B7.1/B7.2 interaction between T cell and target cell (91). Subsequently, the logical conclusion by the early CAR pioneers was the integration of a coactivating domain into the signaling moiety of CAR constructs (**Figure 1**), which became a common feature of second generation CARs (92, 93). This resulted in two basic CAR designs that are still relevant for cancer treatment, one using CD28 and a second using 4-1BB as a coactivating domain. In preclinical studies effector T cells expressing CARs harboring a CD28 costimulatory domain have repeatedly shown higher proliferation rates and released higher quantities of the cytokines IL-2, IFN-gamma, and TNF-alpha, than T cells expressing 4-1BB-costimulated CARs (94–96). Interestingly, preclinical data showed, that the CD28zeta format has also a positive effect on regulatory T cells in the tumor microenvironment because of its high capacity to induce IL-2 secretion (97). Hence, this format might be the favored one in the field to create highly active CAR Tregs. Subsequently, the question arose whether the integration of an additional costimulatory domain could have a positive impact on CAR T-cell efficacy, especially since the problem of tumor microenvironment-mediated T-cell exhaustion in cancer treatment became more and more evident. However, third-generation CARs containing an scFv, a CD3zeta domain and two costimulatory domains in tandem (**Figure 1**) have been tested in clinical trials involving small cohorts of patients but, thus far, they have not been associated with enhanced anti-tumor activity to that of second-generation CARs (98). Despite a promising development of CAR T cell-based immunotherapy, B cell malignancies such as ALL or DLBCL, most attempts to induce long-lasting anti-tumor effects with second-generation CAR constructs in solid tumors were less successful (99, 100). In order to enhance the efficacy of redirected T cells in solid tumors CAR T cells were genetically modified to release a transgenic cytokine upon CAR signaling in the targeted tumor tissue (101). The TRUCK strategy (“**T** cells **R**edirected for Antigen-**U**nrestricted **C**ytokine-initiated **K**illing”), also called “4th Generation” (**Figure 1**) unites the direct anti-tumor attack of the CAR T cell with the tumor microenvironment-modulating capabilities of a proinflammatory cytokine. Binding to the CAR cognate antigen on the tumor cell induces NFAT phosphorylation, migration to the nucleus and induction of the NFAT-responsive/IL-2 minimal promoter that drives transgene expression (101). TRUCKs could also potentially be of interest in autoimmune diseases, since the proinflammatory payload could be replaced by an anti-inflammatory cytokine such as IL-10 or TGF-beta. Interestingly, contrary to CD4 expressing helper T cells, activated NFAT1 in Treg cells forms a ternary complex with FOXP3 at the *IL2* promoter that replaces AP-1 (Jun/Fos) in the NFAT : AP-1 complex present in effector T cells (102, 103). FOXP3 thus transforms a transcriptionally activating NFAT : AP-1 complex in effector T cells into a repressive NFAT : FOXP3 complex in regulatory T cells (103). The fifth generation of CARs (**Figure 1**), is also based on the second generation of CARs, with the addition of intracellular domains of cytokine receptors. In this context, Kagoya and colleagues presented a CD19-specific CAR construct containing a truncated cytoplasmic domain from the interleukin IL-2R beta-chain (IL-2Rbeta) and a STAT3-binding tyrosine-X-X-glutamine (YXXQ) motif that was incorporated into CD28-CD3zeta activation moiety (104).

## Antigen-specific regulatory T cells

As demonstrated by various groups, antigen-specific Treg cells showed higher potency in immunosuppression than polyclonal T cells in diverse preclinical mouse models (105–107). However, the low Treg cell frequency often limits the expansion of endogenous antigen-specific Treg cells and hence prevent their therapeutic use. For this reason, in recent years technical solutions have been sought to generate antigen-specific Treg cells in sufficient quantities and qualities. Promising current methods include redirecting regulatory T cells using synthetic receptors on the one hand and converting antigen-specific effector T cells into regulatory T cells using FOXP3 overexpression on the other hand. Although both CAR and T cell receptor (TCR) based constructs are available for Treg redirection, CAR constructs are mainly used. Unlike MHC-restricted TCR targets, potential antigens recognized by CARs also include non-protein targets such as carbohydrates and glycolipid molecules (108, 109). However, we must also note that CAR constructs only recognize and target cell surface antigens, while TCR constructs can target both MHC-restricted cell surface and intracellular antigens. Moreover, CAR redirected T cells reveal promising results in the treatment of hematological malignancies (110) but demonstrate little effects on solid tumors (111), while T cells engineered to express TCR constructs display encouraging curative outcomes in the therapy of solid tumors (112). Some preclinical studies suggest that the antigen density recognized by the CAR must be high on the target cell to initiate T cell activation (113, 114). Additionally, cross-presentation of the antigen in nearby/draining lymph nodes might be an important factor for TCR redirected cells to build up a reservoir and maintain activation (115). Therefore, the use of TCR redirected T cells could be appropriate for antigens expressed in low densities whereas T cells modified to display CAR constructs better target overexpressed antigens. A safety problem of the TCR Treg cell strategy is also caused by potential TCR mispairing with the endogenous TCR resulting in T cells with unpredicted specificity. To avoid this, replacement of the endogenous TCR by using gene editing technology as recently reported by Stadtmauer might be necessary (116). Finally, although no cytotoxicity was demonstrated in the initial preclinical CAR-Treg cell studies, first Macdonald and then Boroughs and colleagues reported that CAR-stimulated Tregs might also exhibit cytotoxic activity (57, 117). Thus, the cytotoxic activity of CAR Treg cells needs to be studied in more detail to avoid unexpected adverse effects in the future. The very first preclinical study with antigen-specific CAR redirected Tregs was already performed in 2008 by the Eshhar’s group. As demonstrated by the authors, CAR redirected Treg cells accumulated at colonic inflammatory lesions and suppressed effector T cells in a specific, non-MHC-restricted manner, resulting in significant amelioration of colitis (55). Interestingly, the authors were already using a second-generation CAR containing a CD28-gamma signaling domain. The CAR design has changed only slightly until today, whereby the gamma-signaling unit was replaced by the CD3zeta signaling domain. In this context, Dawson and colleagues reported a comprehensive comparison of coreceptor signaling domain CAR variants in human Treg cells and revealed that inclusion of the CD28 costimulatory domain was essential for potent function (28). Moreover, CARs encoding domains from TNFR family members, such as 4-1BB, were unable to confer a protective effect in comparison to irrelevant Ag-specific control Treg cells (28). As shown by Lamarthée and colleagues, ligand-independent CAR tonic signaling significantly affects the biology of CAR-Tregs and thereby compromises their suppressive function (118). The authors demonstrated in their study that the negative effects of 4-1BB tonic signaling in Treg cells could be mitigated by transient mTOR inhibition (118). Currently, the range of applications using redirected Treg cells has been expanded to other diseases such as Graft versus hosed disease (GVHD), type 1 diabetes, multiple sclerosis, vitiligo, asthma or haemophilia (27, 56, 119–122) (see also **Table 1**). Based on preclinical but also clinical results, we can already notice today that CAR Treg cells are slowly emerging as a promising strategy for the treatment of autoimmune diseases and as adjunctive therapy in transplantation. Many clinical studies were already performed using polyclonal Treg cells (19, 20, 22, 26, 123, 124) with important lessons learned from, e.g. production procedures, Treg stability, cell doses, circulation in patients, tolerability and combination with immunosuppressive drugs. Some clinical efficacy could be seen by successful weaning of immunosuppressives or, for diabetes, prolonged c-peptide production. However, the suppressive activity at the site of inflammation could not be detected and there was no improvement in metabolic function or rejection rate, respectively. Preclinical data indicate that engineered Treg cells (CAR or TCR) might be more efficacious to treat autoimmune diseases and transplantation rejection, but the clinical use of redirected (CAR) Treg cells is only just beginning (**Table 1**). The purpose of the first in human study with redirected CAR Treg cells is to evaluate the safety and tolerability of HLA-A2-specific CAR Treg cells (TX200-TR101) and its effects on the donated kidney in living donor kidney transplant recipients (17). There is another HLA-A2-specific CAR Treg study (LIBERATE, Phase I/II) starting in 2022, which, most interestingly, will also address clinical outcome and immunosuppression in liver transplantation (QEL-001) (18). In this context, HLA-A2-specific CAR redirected (CD4 or CD8) Treg cells have been already used in different pre-clinical studies of skin transplantation demonstrating superior suppression of human skin graft rejection and reduced GvHD in humanized mouse models (58, 125, 126).

## Discussion

Although the immunomodulatory properties of Treg cells in the field of autoimmune diseases and transplantation medicine has been repeatedly demonstrated in many preclinical studies and clinical trials, some limitations have prevented the widespread use of this form of immunotherapy. This includes, on the one hand, the selection of the suitable Treg cell population to enhance efficacy. On the other hand, the targeted immunosuppressive effect on the local immune response is not supposed to cause global immunosuppression. Recently, technical progress such as redirection of T cells by CAR constructs, conversion of T cell into Treg cell using *FOXP3* transfer or targeted gene editing using CRISPR/Cas9 allow to design Treg cells with a defined specificity and functionality in a rapid and efficient manner. New applications of Treg cells outside autoimmune diseases and transplantations demonstrate multiple uses of Treg-mediated immune modulation. As demonstrated by numerous groups, in many injured tissues, so-called ‘repair’ Treg cells are recruited to the damaged site to facilitate inflammation resolution and to regulate immunity after injury (127–129). Furthermore, Baek and colleagues demonstrated that Treg cell administration has a neuroprotective effect on pathology and cognitive function in a mouse model of Alzheimer’s disease. In detail, Treg cells had an impact on cognitive function, decreasing amyloid-beta deposition and inflammatory cytokine levels (130). In the future, the use of redirected CAR-Treg cells can potentially increase the efficacy and prevent global immunosuppression of Treg-based immunotherapies and thus make an important contribution to clinical implementation (17, 18), giving new hope for a cure to millions of suffering patients.

## Author contributions

MC and TR participated in manuscript writing, editing and cooperation of its submission. All authors contributed to the article and approved the submitted version.

## Funding

The authors received funding from Deutsche Krebshilfe (DKH) grant 3641 0237 21 and Deutsche Forschungsgemeinschaft (DFG) grant CH 2463/1-1. This work was further supported by the SFB1530.

## Conflict of interest

The authors declare that the research was conducted in the absence of any commercial or financial relationships that could be construed as a potential conflict of interest.

## Publisher’s note

All claims expressed in this article are solely those of the authors and do not necessarily represent those of their affiliated organizations, or those of the publisher, the editors and the reviewers. Any product that may be evaluated in this article, or claim that may be made by its manufacturer, is not guaranteed or endorsed by the publisher.
